# Predicting Brain Age at Slice Level: Convolutional Neural Networks and Consequences for Interpretability

**DOI:** 10.3389/fpsyt.2021.598518

**Published:** 2021-02-25

**Authors:** Pedro L. Ballester, Laura Tomaz da Silva, Matheus Marcon, Nathalia Bianchini Esper, Benicio N. Frey, Augusto Buchweitz, Felipe Meneguzzi

**Affiliations:** ^1^Neuroscience Graduate Program, McMaster University, Hamilton, ON, Canada; ^2^School of Technology, Pontifical Catholic University of Rio Grande do Sul (PUCRS), Porto Alegre, Brazil; ^3^BRAINS - Brain Institute of Rio Grande do Sul, Porto Alegre, Brazil; ^4^Graduate School of Medicine, School of Medicine, Pontifical Catholic University of Rio Grande do Sul (PUCRS), Porto Alegre, Brazil; ^5^Mood Disorders Program, Department of Psychiatry and Behavioural Neurosciences, McMaster University, Hamilton, ON, Canada; ^6^Women's Health Concerns Clinic, St. Joseph's Healthcare, Hamilton, ON, Canada; ^7^Graduate School of Psychology, School of Health and Life Sciences, Pontifical Catholic University of Rio Grande do Sul (PUCRS), Porto Alegre, Brazil

**Keywords:** brain age, deep learning, neuroimaging, convolutional neural networks, model interpretability

## Abstract

**Problem:** Chronological aging in later life is associated with brain degeneration processes and increased risk for disease such as stroke and dementia. With a worldwide tendency of aging populations and increased longevity, mental health, and psychiatric research have paid increasing attention to understanding brain-related changes of aging. Recent findings suggest there is a *brain age gap* (a difference between chronological age and brain age predicted by brain imaging indices); the magnitude of the gap may indicate early onset of brain aging processes and disease. Artificial intelligence has allowed for a narrowing of the gap in chronological and predicted brain age. However, the factors that drive model predictions of brain age are still unknown, and there is not much about these factors that can be gleaned from the black-box nature of machine learning models. The goal of the present study was to test a brain age regression approach that is more amenable to interpretation by researchers and clinicians.

**Methods:** Using convolutional neural networks we trained multiple regressor models to predict brain age based on single slices of magnetic resonance imaging, which included gray matter- or white matter-segmented inputs. We evaluated the trained models in all brain image slices to generate a final prediction of brain age. Unlike whole-brain approaches to classification, the slice-level predictions allows for the identification of which brain slices and associated regions have the largest difference between chronological and neuroimaging-derived brain age. We also evaluated how model predictions were influenced by slice index and plane, participant age and sex, and MRI data collection site.

**Results:** The results show, first, that the specific slice used for prediction affects prediction error (i.e., difference between chronological age and neuroimaging-derived brain age); second, the MRI site-stratified separation of training and test sets removed site effects and also minimized sex effects; third, the choice of MRI slice plane influences the overall error of the model.

**Conclusion:** Compared to whole brain-based predictive models of neuroimaging-derived brain age, slice-based approach improves the interpretability and therefore the reliability of the prediction of brain age using MRI data.

## 1. Introduction

Brain age prediction involves estimating chronological age based on information typically gleaned from neuroimaging data. The prediction may be referred to as the biological or neuroanatomical age of the brain. Although brain age can be computed from other approaches, such as the epigenetic clock from brain tissue (1), in this paper we use brain age as a synonym for neuroimaging-derived brain age. The difference between the predicted age and the actual chronological age is called *brain age gap*, which has been associated with a number of lifestyle factors (2) [e.g., tobacco and alcohol consumption (3), obesity (4), diabetes, schooling, physical activity (5), higher mortality risk (6), lower fluid intelligence, psychiatric disorders (7), and neurological diseases (8)].

Recent advances in machine learning, specifically on deep convolutional neural networks, have gradually improved brain age prediction by lowering prediction error (9). However, brain age prediction methods still receive criticism due to the lack of interpretability (10). The criticism stems from the limited information about what the model uses to predict brain age, and which regions might bias findings. Hidden biases and poor generalization are a recurrent theme in machine learning and deep learning research (11), including its medical imaging applications (12). Thus to fulfill the promise of translational research, AI needs to establish reliable and reproducible prediction methods, and to generate models that are more amenable to clinical interpretation (10). Identification of clinical neural markers and association with clinical and behavioral data may render AI applications more meaningful (10, 13, 14).

In this article, we report on a model developed for the PAC-2019 brain age prediction competition. Our goal was to generate competitive predictions using meaningful neuroanatomical information. We developed a deep learning framework whose predictions draw on features from every single slice of brain imaging combined with average or linear regression models. The resulting model associates each slice with an independent age prediction for the same patient, allowing researchers to scrutinize the areas of the brain responsible for the overall brain-age gap. Our hypothesis was that our approach would help understand the behavior of brain age prediction at each part of the brain.We also believe that this method, alongside other approaches that try to move away from single predictions of brain age (16), may help us get a comprehensive picture of the parts and characteristics of the aging brain that inform prediction. Such picture should allow for identification of diverse, slice-level, and eventually voxel-level, neuroanatomical traits of age-related diseases.

## 2. Background

The known patterns of brain development associated with aging, such as a decline in gray matter volume (17), are readily identifiable by magnetic resonance imaging (MRI). These images are extensively used for diagnostic and research of disorders associated with brain tissue loss, such as Alzheimer's disease, Parkinsonian dementias, and Fronto-temporal lobe degeneration (18). More recently, machine learning techniques have been used to draw on the rich MR images to predict the brain age of healthy people (19), and the aging processes of neurodegenerative disorders (20). The mismatch in chronological and brain age has been investigated in schizophrenia (8), bipolar disorder (21), and in association with factors associated with mortality risk, physical and mental fitness, and biological health (6).

Recent advances in deep learning models, specifically Convolutional Neural Networks (CNNs) achieve state-of-the-art performance in computer vision tasks (22), while requiring little to no prior hand-engineering of data. CNN architectures using 3D convolutions have been used to predict brain age with segmented GM and white matter (WM), and raw T1-weighted MRI scans (10, 23). The use of 3D convolutions allows the model to take in whole-volume information for convolutional filtering operations, which, given enough data, learn feature detection and extraction. CNN models provide highly accurate predictions for regression and classification tasks on multiple medical imaging datasets (22, 24).

CNN models have remarkable predictive power, but the results are typically difficult to interpret. Whereas manual feature selection in classic machine learning simplifies interpretation of the model's results, CNNs require further processing steps to interpret the model's decision processes due to the use of less processed data (15). Examples of interpretation-seeking mechanisms include saliency maps (25) and activation mappings (26), which aim to identify the regions in an image that are responsible for assisting model predictions, thus allowing for some visualization of key input features. These maps trace network outputs back to the input image voxels through the computation of their partial derivatives. For example, regression activation mapping applied to age prediction models on newborn structural MRI generated brain maps of rapid growth during early development (27). Saliency maps have three key limitations. First, they depend on human validation, a time consuming task that also entails the potential for confirmation bias. a Second, these methods can produce results that are independent of model and data, and thus inadequate for model debugging and inspection (28). Finally, additional techniques must be used for combining individual subject saliency maps of into population-level visualizations (29).

## 3. Method

**Figure 1:**
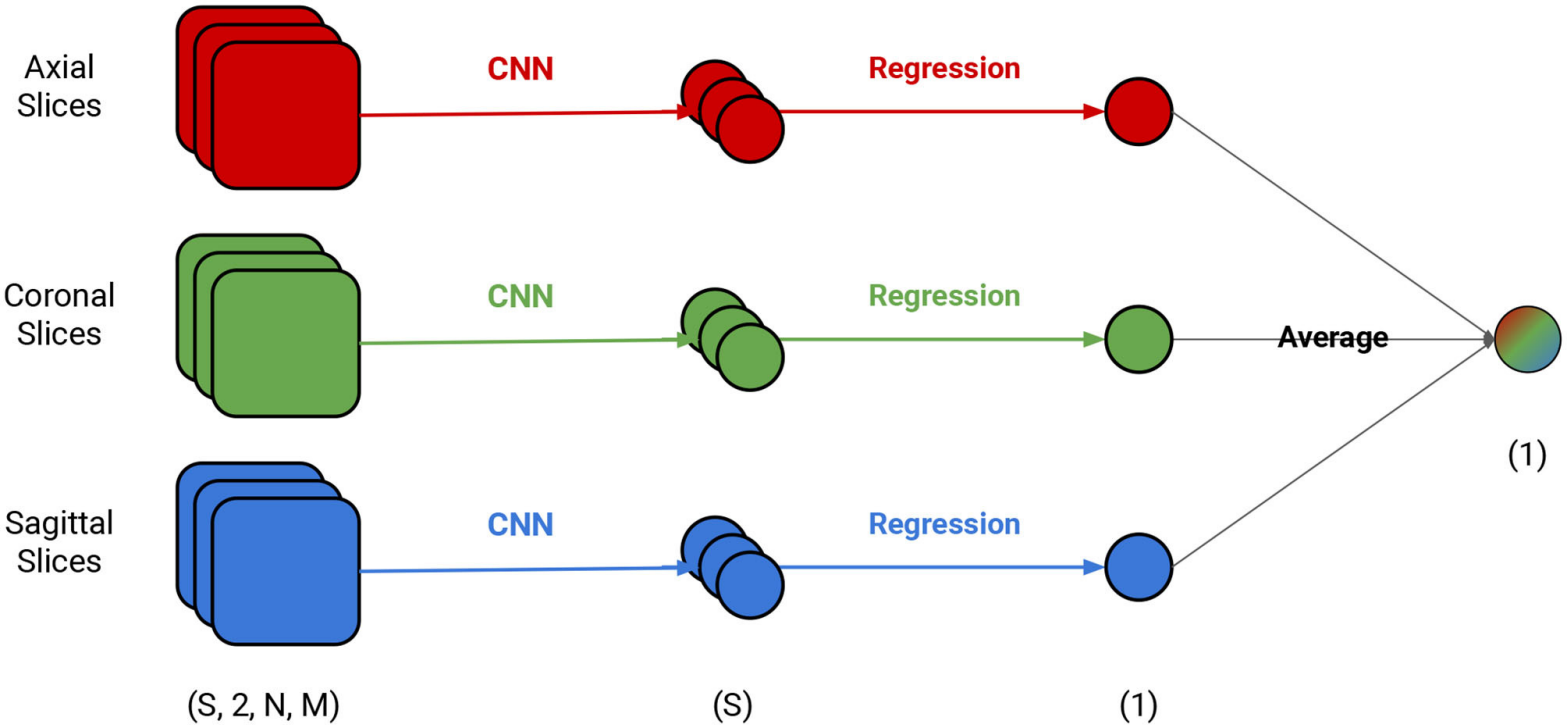
Depiction of the brain age prediction framework. Each view has an independent CNN model and an independently-trained linear regression model. *S* is the number of slices and *N* and *M* are the dimensions of the slice (e.g., if evaluating the axial slice, the *N* and *M* are the dimensions for the sagittal and coronal views).

We tested several models and found that the ones with a RESNET18 architecture had a good trade-off between size and prediction error (30). In order to use it in our context with the dimensions of our input, we modified it in three simple ways: (1) the input size had one or two channels, depending on the experiment, (2) the kernel size from the average pool was changed from 7 to 4, (3) the final fully connected layer was changed from 512 to 1,024. The code to build this architecture as well as the steps to reproduce all experiments are available on GitHub (see data availability statement). The input was one brain slice with segmented GM in the first channel and white matter in the second channel. The segmentation was provided by PHOTON-AI ^1^ and we made no adjustments to it apart from scaling. The output of each model was a brain age estimation for a single segmented slice (GM, WM, or both) from the structural *MRI*. We illustrate our framework in Figure 1. The framework relies on three different, simultaneously-run models that are combined by three linear regression models and a final average. Each model is trained independently to predict brain age from a single MRI slice and draws on different MRI slice orientation (coronal, sagittal, or axial) as input. Each of these models used the validation set to generate error estimates for each slice and each volume. We then used the error estimates to determine the importance of each slice for the model. All three views were combined to locate specific regions in the brain responsible for a particular classification based on the contribution of each slice for the brain age prediction, which provides an additional source of interpretation.

The final age prediction for a single individual was calculated as follows:

(1) $$\begin{array}{l} a_{x}=M_{x}\left(s^{i}\right)i\;\in \;\left[0,S_{x}\right] \end{array}$$

(2) $$\begin{array}{l} age=\frac{\frac{1}{e_{a}}*L_{a}\left(a_{a}\right)+\frac{1}{e_{s}}*L_{s}\left(a_{s}\right)+\frac{1}{e_{c}}*L_{c}\left(a_{c}\right)}{\frac{1}{e_{a}}+\frac{1}{e_{s}}+\frac{1}{e_{c}}} \end{array}$$

where *x* ∈ *a, s, c* represents the axial, sagittal, and coronal views, *a*_*x*_ is the age vector for each slice for the subject, *M*_*x*_ is one of the CNN models, *L*_*x*_ is the linear regression model, *e*_*x*_ is the error of model *M*_*x*_ in the validation set, and *S*_*x*_ is the total number of slices for an orientation *x*. Our rationale was that each model's contribution was inversely proportional to the error in the validation set with a weighted average. The influence of each slice for the final prediction is weighted by the linear regression model. This slice-level rationale can also be applied to understand the independent contributions of gray and white matter to the brain age estimate. While using gray or white matter alone causes the model to lose predictive power, it improves interpretability by estimating the independent gray and white matter contributions to the age prediction.

All segmented MRI scans for the dataset provided by the competition were shaped (121, 145, 121). To reduce the amount of empty space on the corners of the input, we removed 20% of the image corners, resulting in a (72, 88, 72) image. The input for the model is thus shaped (*batch, c*, 72, 88) where *c* = 1 when using either gray or white matter alone or *c* = 2 when using both types of brain tissue. For the coronal view, we zero-pad the image so that the (72, 72) slice also becomes (72, 88) to keep the consistency across all models. The participants from the PHOTON-AI dataset included healthy individuals from a wide age range, males and females, and from 17 different centers. We included basic demographic information about the sample in Table 1.

**Table 1:** Participants information.

**Center**	**Age mean (std)**	**Sex (F-M)**
0	34.24 (12.67)	197-133
1	26.76 (9.23)	79-55
2	35.51 (12.18)	331-244
3	25.76 (6.62)	18-129
4	21.24 (2.01)	85-58
5	31.25 (7.46)	21-18
6	62.70 (6.75)	3-7
7	43.44 (11.27)	15-10
8	24.82 (5.16)	121-137
9	49.13 (16.62)	255-194
10	33.19 (11.34)	23-51
11	69.92 (7.97)	9-9
12	28.77 (7.77)	16-15
13	41.00 (17.80)	52-76
14	44.41 (22.81)	142-88
15	23.27 (1.27)	16-3
16	22.76 (2.80)	20-9
Total	35.88 (16.21)	1,403–1,236

**Figure 2:**
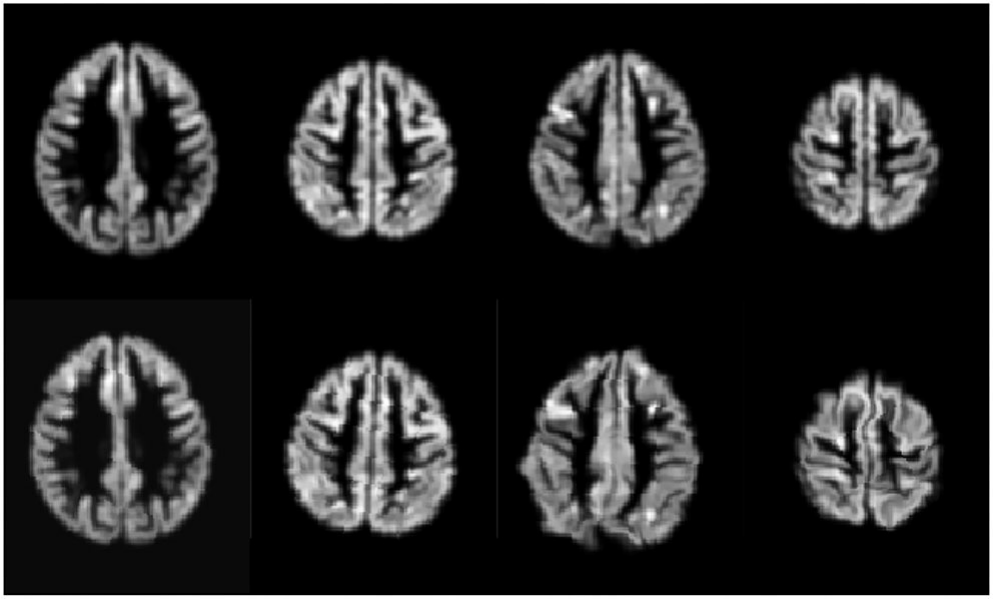
Examples of the augmentation procedure. First row are gray matter segmented images before augmentation; the second row are their augmented counterparts.

We trained the CNN models using an Adam Optimizer set with the learning rate at 6*e* − 4 and weight decay of 6*e* − 4. The training also used a sigmoid learning rate rampup for 20 epochs followed by a cosine rampdown until a total of 100 epochs. The batch size was set to 64. We conducted a data augmentation with Elastic Transform (31) with an α range between [28, 30], σ with a range of [3.5, 4.0], and *p* = 0.3, representing the scaling factors, the Gaussian spatial smoothing of the deformation field, and the probability of the augmentation being applied.; Random Affine transformations with 4.6 degrees, [0.98, 1.02] scale, and translation of 0.03; finally, we used a Random Tensor Channel Shift with the range of [−0.1, 0.1]. Some examples of the augmentation procedure can be seen in Figure 2. All data augmentation procedures were implemented in the medicaltorch framework^2^.

## 4. Results

For the competition, we achieved a mean absolute error of 4.44 years on the test set, with a Spearman correlation of −0.25 between the age estimates and chronological age. The model that won the competition achieved 2.90. Due to time restrictions, we employed axial slice predictions only, combining gray and white matter. In what follows, we present the results for the combined gray and white matter models for each separate orientation, the combined predictions; we also present how predictions improve interpretability and decrease model errors. The results of this article are based on predictions made on the competition's validation set. We did not have access to the test set's ground truth at any point during our experiments, prior to nor after the competition.

Instead of using the validation set to train the linear regression, we applied the regression to the training set, in order to avoid circular analysis. However, we believe this can limit the accuracy that otherwise would be achievable with the linear regression model. We leave the comparison of using the validation dataset and reusing the training set to train the linear regression model for future work with more data available, as we restricted ourselves to use data exclusively from the challenge for this study.

### 4.1. Combined Gray and White Matter

Our approach used slices for both gray and white matter in individual channels. The use of the two tissues simultaneously may have sacrificed obtaining more fine-grained information about brain aging from the each independent tissue, but it was done in favor of feeding additional data to the model. Gray and white matter were concatenated on the first channel, resulting in an input of (2, 72, 88). We then trained and evaluated the model, and then assessed the effects of age, sex, and site on its predictions. In the sections, we explain the key findings from our experimental analysis.

#### 4.1.1. Models for Different Views of the Brain Have Different Errors

We trained three independent models, taking the input from either axial, coronal, or sagittal views (Note: for the competition, in the interest of time, we used only the axial orientation information). After training, we estimated the error for each slice. The estimate is used to gauge how much each slice contributes to the prediction of brain age.

**Figure 3:**
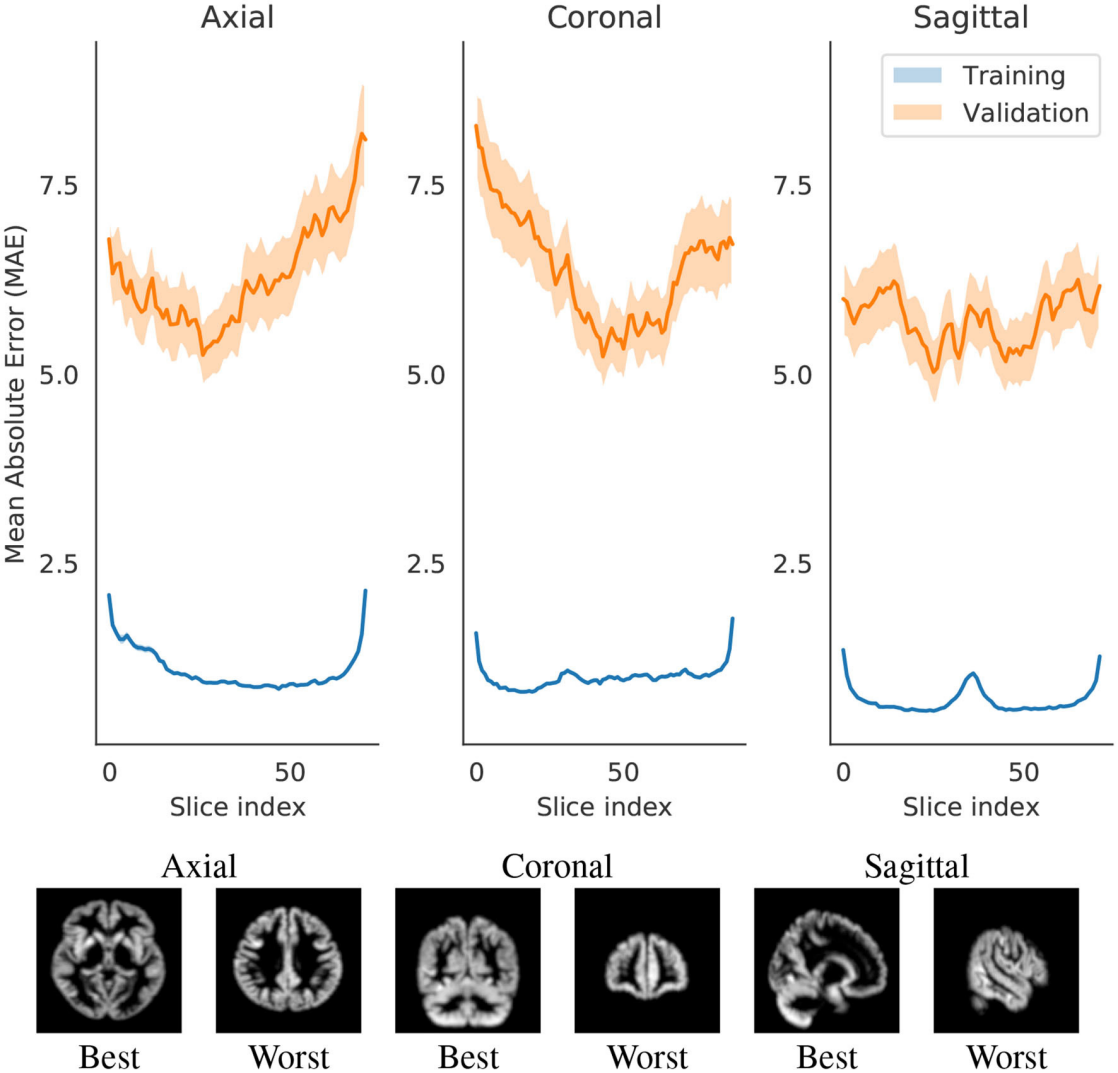
Change in Mean Absolute Error (MAE) with respect to changing the slice that is evaluated by the network. Each slice index value are an average of either all training set or all validation set. The shade represents the 0.95 confidence interval for those points. The slices in the image are examples of the index that best or worst predicts brain age.

We identified a pattern, most present in axial and coronal models, but to some extent also visible in the sagittal. With more distal slices, the average prediction error seems to be higher. This could arguably be attributed to several reasons: (1) differences of brain matter across regions of the brain, (2) more age-related changes in some regions than others, and (3) the tendency of noise from the scanner to be concentrated on the extremities of the image (and this tendency is fairly visible in Figure 3).

The three models afforded different final prediction errors (Table 2, which we will further discuss in section 4.1.2. The models also resulted in different estimates for the contribution of slices for predicting brain age. Figure 3 shows the error variation for slices for the axial, coronal and sagittal slice models. We hypothesize that the differences in mean error may stem from: intrinsic and extrinsic factors that contributed to a poorer segmentation (and therefore an input of lesser quality); the randomness of the modeling process; and from sample heterogeneity for each of the regions with respect to age.

**Table 2:** Final validation results for all views.

**View**	**Average slice error**	**Average error**	**Regression error**	**R^2^**
Axial	6.28	4.88	5.09	0.82
Coronal	6.38	4.91	5.04	0.83
Sagittal	**5.71**	**4.45**	**4.52**	**0.86**
Combined			4.62	0.86

*The Average Slice Error column evaluates the final error of our model by doing a simple average across all slices of the volume. The Average Error column uses the same information for the slice column, but instead of calculating the average error for each slice, calculates the mean prediction of all slices, and compare with the actual age. The Regression Error column is the error of using linear regression instead of an average to classify the whole volume. All values are mean absolute errors*.

#### 4.1.2. Final Predictions

Using Equations (1) and (2), we generated the predictions for the final dataset. After pre-training each of three models (one for each view), we evaluated every slice in the dataset and generated a dataset of predictions with a row for each individual and a column for model predictions of every slice. We trained the linear regression for the generated dataset using scikit-learn ^3^ library.

Table 2 shows the results for the three models. The sagittal slice prediction showed the lowest error, and it outperformed the outputs combined using Equation (2). We argue that this discrepancy in the error is due to the lack of another validation set for proper out-of-sample error estimation. Additionally, the differences present in a model trained with this dataset may not accurately translate to other data sources. To ensure that the sagittal slice prediction is actually superior to the axial and coronal slice predictions, the model needs to be further validated and trained using yet another dataset.

#### 4.1.3. Age Effects

Brain age prediction methods usually perform better around the mean chronological age of the dataset. Research shows that models tend to overestimate brain age for participants younger than the mean, and underestimate it for participants older than the mean (32, 33).

**Figure 4:**
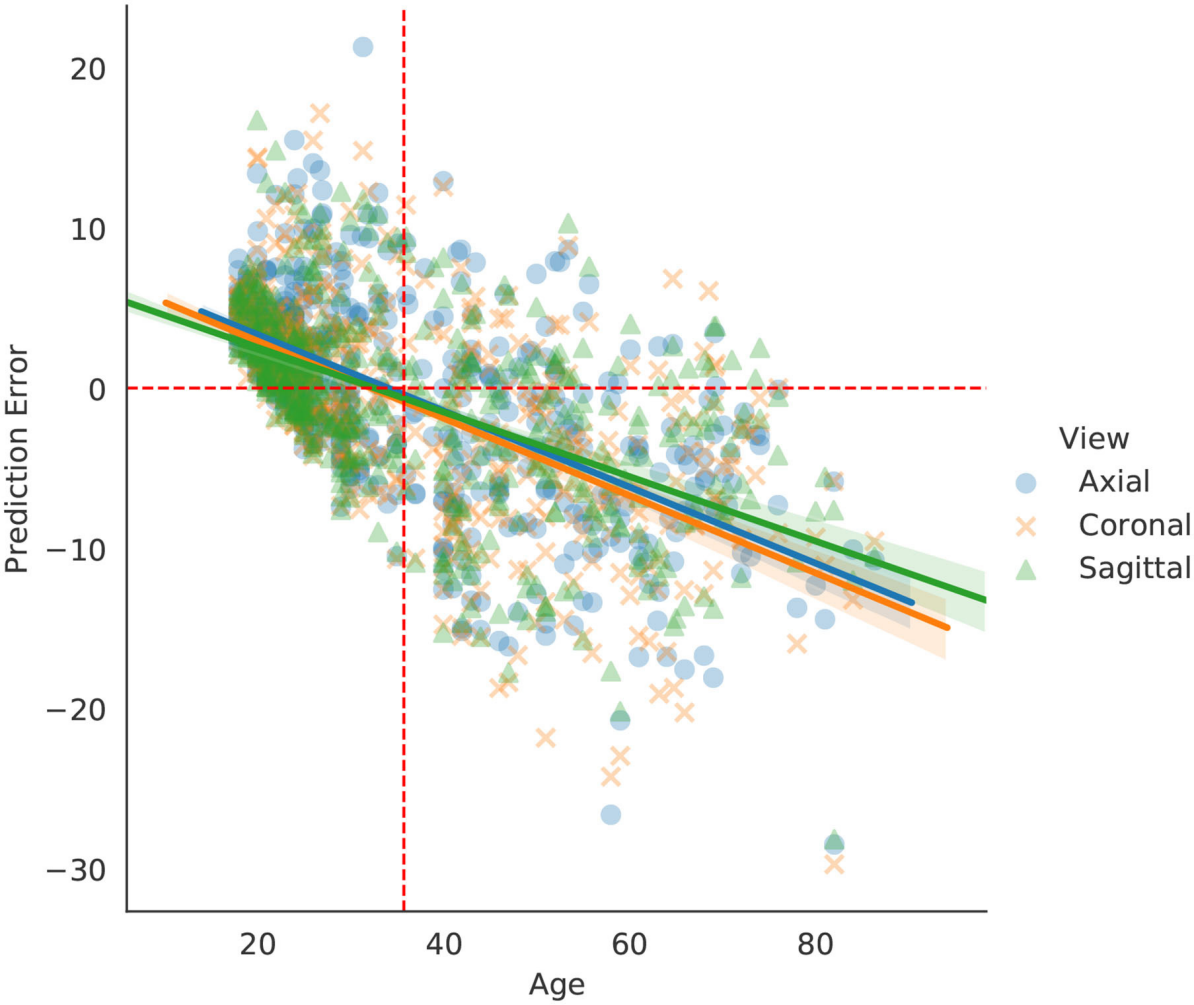
Regression curves for the validation set. Every point represents a person (each person is presented three times, one for each view). Dashed red orthogonal to the x-axis is the age average of the dataset, while the horizontal dashed line is aligned to 0 error as a reference.

The behavior of our model's prediction error changed with respect to age, as shown in Figure 4. Not surprisingly, we found the same pattern of over and underestimation of age reported in the literature. One major implication of this type of error is the possibility of inserting biases when trained models are applied to external test sets that have a different age distribution than the training set. Thus, brain age prediction models should consider or aim for age-matched test sets (if possible), even if the model has been validated in external datasets. That way, if any underestimation or overestimation of age is happening with the target set, it can be identified and properly dealt with before any conclusions are drawn. Other means of mitigating the effect of testing models on datasets with different age ranges has been addressed elsewhere (33, 34).

#### 4.1.4. Site Effects

**Figure 5:**
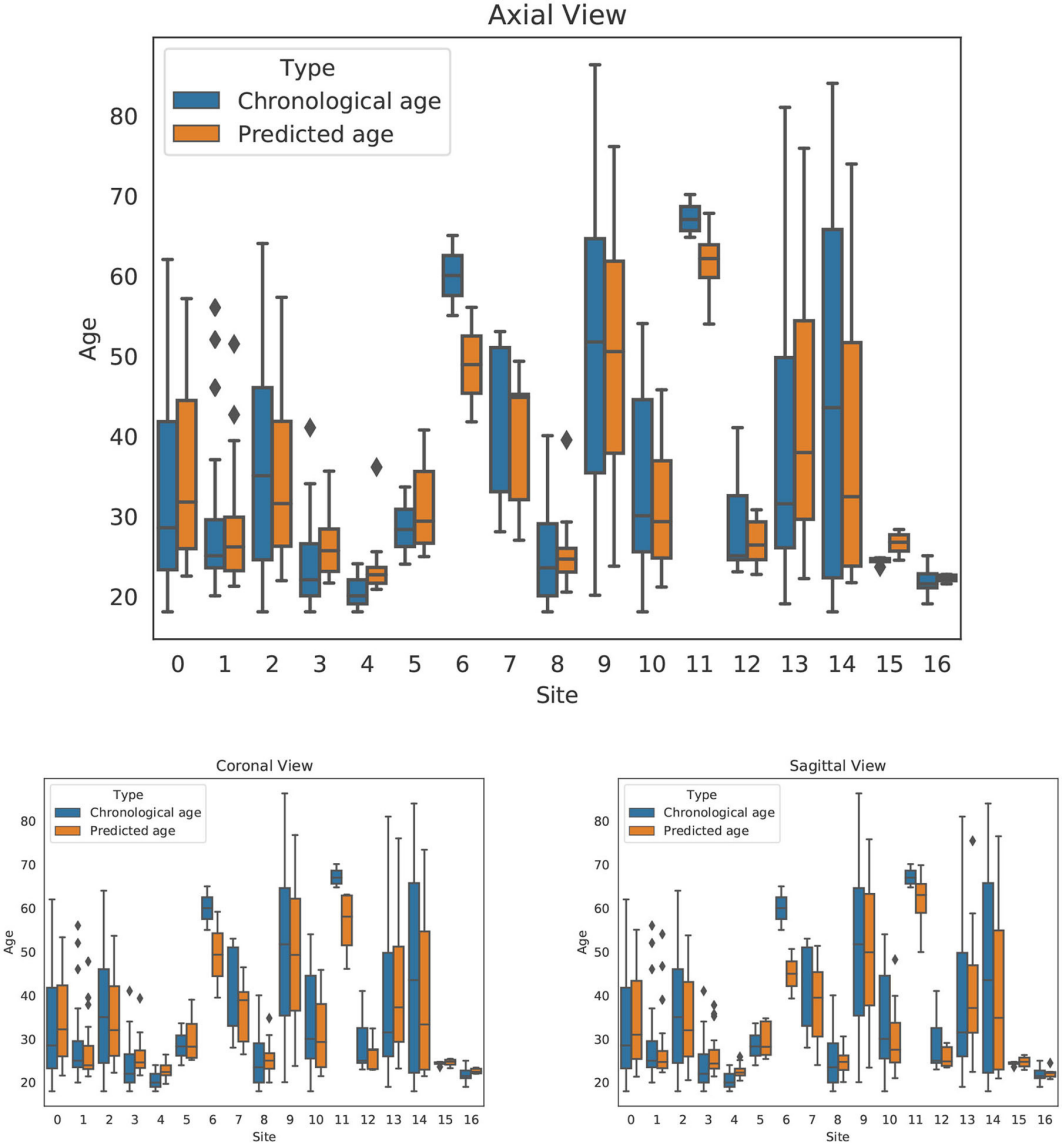
Site effects for axial, coronal, and sagittal views. For each orientation, the chronological age and predicted age are shown side-by-side by site.

The dataset included contributions from 17 different sites. To investigate any biases associated with the different sites, we extracted the age and predicted age for each of our models and each of the 17 sites. We ran a two-tailed dependent *t*-test to compare age and predicted age among sites and found statistically significant differences for just 6 out of the 17 centers. The data are presented in Table 3 and Figure 5. In four out of the six significantly different centers, all views had significant differences simultaneously, thus suggesting that the models tend to operate in similar manner, and that an actual superiority of the sagittal slice model needs to be further investigated.

**Table 3:** Site effects for each orientation and each site. *p* < 0.03 in bold.

**Site**	**View**	**t-Statistic**	**p-value**
0	Axial	−2.825	**0.006**
	Coronal	−1.581	0.119
	Sagittal	−1.985	0.051
1	Axial	0.829	0.415
	Coronal	1.904	0.068
	Sagittal	1.788	0.085
2	Axial	3.248	**0.002**
	Coronal	3.248	**0.002**
	Sagittal	3.097	**0.002**
3	Axial	−3.006	**0.005**
	Coronal	−2.335	**0.027**
	Sagittal	−2.845	**0.008**
4	Axial	−5.293	**0.000**
	Coronal	−6.040	**0.000**
	Sagittal	−5.188	**0.000**
5	Axial	−1.945	0.093
	Coronal	−1.111	0.303
	Sagittal	−1.323	0.227
6	Axial	5.178	0.121
	Coronal	2.203	0.271
	Sagittal	22.157	**0.029**
7	Axial	−0.010	0.992
	Coronal	1.080	0.341
	Sagittal	0.529	0.625
8	Axial	−1.436	0.157
	Coronal	−1.921	0.060
	Sagittal	−0.957	0.343
9	Axial	0.698	0.487
	Coronal	1.845	0.068
	Sagittal	0.914	0.363
10	Axial	0.801	0.437
	Coronal	0.961	0.353
	Sagittal	1.675	0.116
11	Axial	3.146	0.051
	Coronal	3.574	0.037
	Sagittal	1.575	0.213
12	Axial	0.873	0.416
	Coronal	1.203	0.274
	Sagittal	1.387	0.215
13	Axial	−1.973	0.060
	Coronal	−1.293	0.208
	Sagittal	−1.296	0.207
14	Axial	4.478	**0.000**
	Coronal	3.960	**0.000**
	Sagittal	4.114	**0.000**
15	Axial	−2.058	0.132
	Coronal	−0.393	0.721
	Sagittal	−0.336	0.759
16	Axial	−0.527	0.621
	Coronal	−0.858	0.430
	Sagittal	−0.418	0.693

#### 4.1.5. Sex Effects

Previous papers suggest that sex can play a role in biasing brain age prediction models. For that reason, we assessed how sex influenced our predictions. We executed a two-tailed paired *t*-test for age and model predictions for both males and females independently and found no significant differences (*p < 0.03*). We ran a two-tailed unpaired *t*-test for males and females to see whether predictions or age was significantly different between groups, also with negative findings (*p < 0.03*). Table 4 summarizes our tests. we applied a two-tailed dependent *t*-test to compare group means, the test showed no significant differences.

**Table 4:** Sex effects for each combination of age and prediction and males and females.

**Group 1**	**Group 2**	**Variable 1**	**Variable 2**	**Test type**	***t***	**p-value**
Sex M	Sex F	Age	Age	I	*t*(531) = −1.957	0.051
Sex M	Sex F	Pred	Pred	I	*t*(531) = −2.088	0.037
Sex M	Sex M	Age	Pred	D	*t*(237) = 0.558	0.578
Sex F	Sex F	Age	Pred	D	*t*(293) = 1.423	0.156

*All tests are two-tailed. Results presented here are for the axial orientation model in the validation set. Values in parentheses are the degrees of freedom for each test. I, independent (or unpaired); D, dependent (or paired); Pred, prediction*.

#### 4.1.6. Voxel-WiseLevel Brain Age Predictions

**Figure 6:**
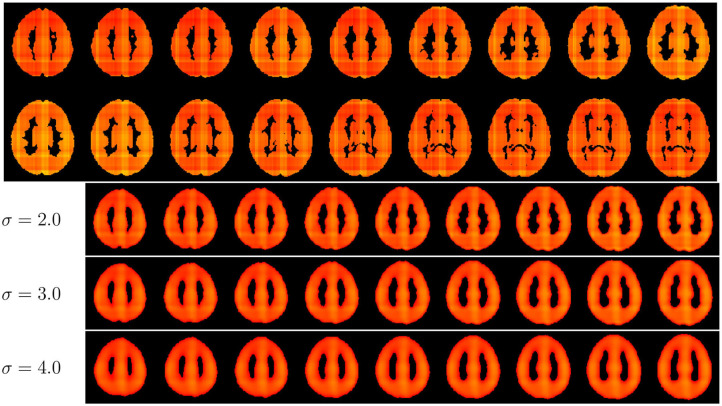
Lightbox view of axial slices age predictions following the voxel-level approach. The value for σ indicates the amount of gaussian spatial smoothing applied to the predictions. Images on a range from 20 (red) to 60 (yellow).

We investigated whether a voxel-level model could benefit from the three slice predictions. For each axial slice, information was gathered from each intersecting coronal and sagittal slice, and the combined with the axial prediction to generate an average for each voxel. We show an example of its use in Figure 6.We believe there is a multitude of applications and benefits that come with this sort of approach, such as: (1) the exploration of distinct brain aging patterns in different regions of the brain; these patterns do not need to be bounded by anatomically or functionally defined regions, (2) the investigation of region-specific brain aging in neuropsychiatric disorders that present a brain age gap, and (3) the identification of voxel- or region-specific prediction biases (e.g., regions that consistently present higher error). But this approach led to unstable predictions, possibly because the model was not being trained using voxel-level information. There were high frequency changes in predictions for neighboring slices; it is expected that neighboring slices be the opposite. This problem has been documented in deep learning patch-based prediction methods (35). A possible simple but sub-optimal solution is to use gaussian spatial smoothing to remove the highfrequency changes, as we demonstrate in Figure 6. We contend that future approaches may aim to develop means to mitigate the artifacts that come “stitching” slice-level predictions into voxel-level ones. Such a successful approach could improve the current model.

### 4.2. Independent Gray and White Matter

**Figure 7:**
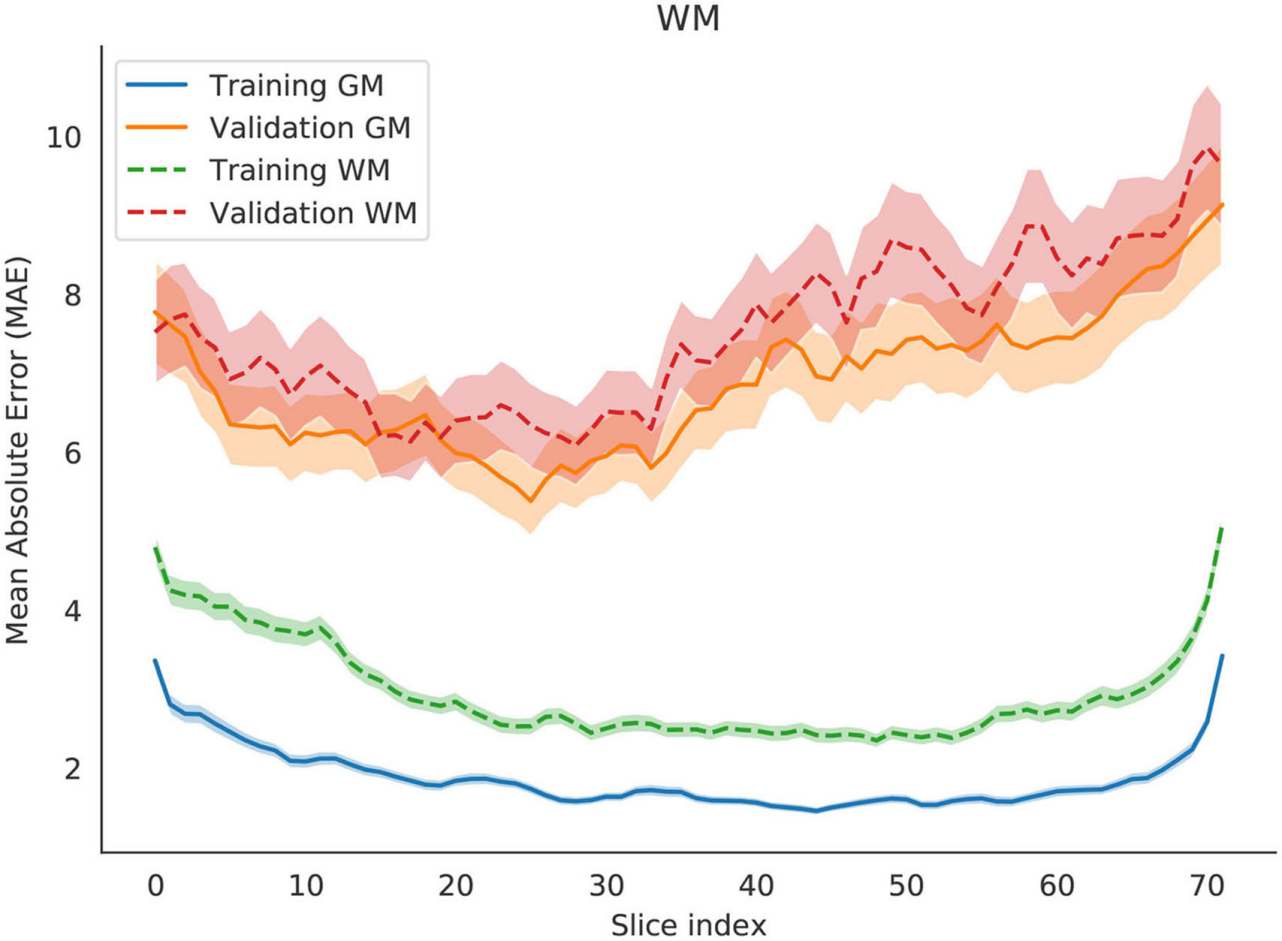
Change in Mean Absolute Error (MAE) with respect to changing the slice that is evaluated by the network. Two independent models are evaluated, one for gray matter (solid lines) and another one for white matter (dashed lines). Each slice index value is an average of the entire training set or the entire validation set. The shade represents the 0.95 confidence interval for those points.

To estimate the contribution of gray and white matter tissue to the prediction, we created another model (for axial slices) to test age predictions using gray matter and white matter separately. Based on the results in Figure 7, the regions that are best predictors of brain age are similar between gray and white matter. The model using solely white matter presented higher errors in most slices. Previous studies have shown that gray matter is a better predictor of brain age than white matter (9).

## 5. Discussion and Conclusion

Identifying the most predictive regions of the brain for brain age models may help interpreting results, but may also introduce model biases that are unrelated to a neurological condition. Our study presents a model that attempted to balance accuracy and interpretability of the results. Our model provides a level of scrutability for the decision-making process, and can thus help researchers and clinicians understand its limitations.

Given the number of perspectives on interpretability for machine learning (36, 37), we must clarify exactly what we mean by interpretability. Although the interpretability or explainability are commonly used to refer to strategies that explain model predictions, we use it more broadly to define that the final predicted brain age can be attributed to specific slices or regions of the brain. In case additional explanation is needed for a single slice, the usual strategies, carrying the limitations we discussed in section 2, should be applied. We believe that for research purposes, knowing the influence of each part of the brain in the final prediction is imperative to guide and interpret findings of research using the brain age gap.

As a whole, neuropsychiatric research on the brain age gap has mostly focused on associating the difference between the chronological and predicted age to clinical populations. Moreover, most of this research was conducted on the assumption that the brain age gap actually encompasses a larger brain-wide phenomenon responsible for accelerated brain aging.

The body of work on the prediction of brain age at specific regions is growing, two contributions of which are of note in comparison to our contribution. First, a preprint paper (37) uses a similar approach to ours, but instead of using slices, they rely on 3d patches that are later combined with averages or linear regression. Second, a recently published approach (38) uses slice-level predictions, but instead of combining it with linear regression, uses a recurrent neural network for the job.

Attention-based, especially Transformers, models, originally purposed for text-based modeling (39), have recently shown to offer substantial improvement on classification accuracy for image-based problems (40). However, at the time we developed our approach, attention models were more popular for text-based than image-based tasks. More importantly, we were not aware of any work with neuroimaging data that had shown substantial improvements using those approaches. Indeed, future work should focus on comparing the explanations we provide in terms of slides to the attention maps generated by attention-based models.

Our experiments corroborate some findings from the field. First, we showed that age effects are significant and should need to be accounted for in predictive models of brain age. Second, our results suggest that proper training and test splits that keep site data proportional may mitigate site effects. Third, gray matter seems to be more predictive of age than white matter. Interestingly, our model had similar performance in both male and female sex, although sex is not explicitly used by the model and no separate models were trained.

Since the competition dataset was preprocessed by segmenting gray and white matter, future work should look at less processed data to try to replicate these results. Differences between the unprocessed and segmented inputs might help us understand the extent that possible segmentation errors may influence the behavior of models of brain age.

## 6. Limitations

The convolutional neural network fails to combine information from different regions of the brain due to its 2D nature. Although aggregated at later stages of our framework, 3D CNNs might be able to capture patterns that our proposed method misses.

We also found that the aggregated information from each slice prediction to form a voxel-level age prediction to be noisy enough to be unusable. Adjacent voxels had usually the same error but occasionally, while changing the slice index, the prediction had a drastic change. This behavior can probably be attributable to several issues, such as lack of regularization for more stable predictions or possible unidentified problems with the segmentation maps.

## Data Availability Statement

Publicly available datasets were analyzed in this study. The code to reproduce our experiments is available at GitHub (https://github.com/lsa-pucrs/pac-2019). Data was provided by PHOTON-AI (https://www.photon-ai.com/explorer) during the PAC-2019 challenge.

## Ethics Statement

Ethical review and approval was not required for the study on human participants in accordance with the local legislation and institutional requirements. Written informed consent for participation was not required for this study in accordance with the national legislation and the institutional requirements.

## Author Contributions

PB, LT, MM, and NE designed the study. PB implemented the framework and ran experiments. FM and AB supervised the implementation and engineering of the work. AB and BF helped interpreting the findings and provided neuroimaging-related insights. All authors contributed to writing the manuscript.

## Conflict of Interest

BF had a research grant from Pfizer outside of this study. The remaining authors declare that the research was conducted in the absence of any commercial or financial relationships that could be construed as a potential conflict of interest.

## Footnotes

^1^https://photon-ai.com/

^2^https://github.com/perone/medicaltorch

^3^https://scikit-learn.org/
